# Sonidegib treatment in patients with locally advanced basal cell carcinoma

**DOI:** 10.1111/dth.15348

**Published:** 2022-02-14

**Authors:** Alessia Villani, Gabriella Fabbrocini, Massimiliano Scalvenzi

**Affiliations:** ^1^ Dermatology Unit, Department of Clinical Medicine and Surgery University of Naples Federico II Naples Italy

Dear Editor,

Advanced basal cell carcinomas (BCCs), including metastatic BCC (mBCC) and locally advanced BCC (laBCC) have always represented a difficult‐to‐treat form of skin cancer; laBCC are primary, recurrent tumors that often progress to an advanced stage requiring treatments different from surgery or radiation therapy.^1^ Hedgehog pathway inhibitors (HHI)s represent a valid therapeutic option for the treatment of advanced BCCs. To date, vismodegib and sonidegib are the two oral drugs approved for the treatment of advanced BCCs^2^; although in literature there are several prospective trials and real‐life studies describing the efficacy and safety of vismodegib in treating advanced BCCs, to date, few data regarding the real‐life experience of sonidegib have been reported.^3^, ^4^ We read with great interest the article written by Leow et al.^5^ presenting a case series of 10 patients with complex BCCs treated with sonidegib, and we also want to report our real‐life experience regarding patients with laBCCs treated with sonidegib. Adult patients with a single laBCC treated with sonidegib, at the approved dosage of 200 mg/daily, for a minimum time of 6 months were included in the study. All patients provided informed consent for publication. Patients presenting multiple primary BCCs and/or patients previously treated with vismodegib were excluded. Demographic (age, sex) and clinical (localization, histological subtype) data were recorded at baseline visit, furthermore, treatment related adverse events (AEs) and their degree of severity were also collected. Thirty‐six patients (24 males and 12 females), with a medium age of 75.8 years (53–96 years) were included in the study; all BCCs (median diameter of 4.2 cm) were histologically confirmed with a 5‐mm punch biopsy. The head and neck region was the site most frequently involved (25/36; 69.4%), followed by the trunk (6/36; 16.7%), the legs (3/36; 8.3%) and by the upper limbs (2/36; 5.6%). Patients received sonidegib for a median time of 6.8 months and 10 patients received the alternate dosing regimen (200 mg every other day) in order to reduce the degree of AEs. 17 (47.2%) patients achieved complete remission of the tumor, 14 (38.9%) patients presented >50% clinical reduction of the laBCC, and 5 (13.9%) reported no response to treatment (<50% of tumor size reduction). Regarding treatment related AEs 32 out of 36 patients (88.9%) experienced at least one AE, with dysgeusia (88%), muscle spasms (76.5%), weight loss (57.2%) and alopecia (43%) being the most represented ones. Moreover, the majority of patients experienced mild–moderate graded (grade 1–2) AEs with alopecia being the AE most frequently presenting in a severe grade (grade 3). Advanced BCCs treatment still represents a challenge for dermatologists; in the last decade HHI have represented an important therapeutic alternative for unresectable BCCs often presenting in old patients with multiple comorbidities.^3^ Their efficacy as neoadjuvant treatment and in association with other therapies has also been discussed^6^; further studies on larger sample size examining long‐term recurrences and survival rates are still required.

## CONFLICT OF INTEREST

None.

## AUTHOR CONTRIBUTIONS

*Idea and design*: Alessia Villani, Gabriella Fabbrocini and Massimiliano Scalvenzi. *Data collection*: Alessia Villani. *Revision of the article*: Alessia Villani, Massimiliano Scalvenzi.

## ETHICAL APPROVAL

Approval of ethical committee was obtained from the University Federico II of Naples, Italy.

## Data Availability

Data of this study are available from the corresponding author upon reasonable request.
